# Application of TLC for Evaluation of the Lipophilicity of Newly Synthetized Esters: Betulin Derivatives

**DOI:** 10.1155/2019/1297659

**Published:** 2019-03-03

**Authors:** Katarzyna Bober, Ewa Bębenek, Stanisław Boryczka

**Affiliations:** ^1^Department of Analytical Chemistry, School of Pharmacy with the Division of Laboratory Medicine, Medical University of Silesia in Katowice, Jagiellońska 4, 41-200 Sosnowiec, Poland; ^2^Department of Organic Chemistry, School of Pharmacy with the Division of Laboratory Medicine, Medical University of Silesia in Katowice, Jagiellońska 4, 41-200 Sosnowiec, Poland

## Abstract

The problem of designing a new drug is nowadays very important for researches representing many branches of science. This work considers the usefulness of the analytical method such as thin-layer chromatography for the lipophilicity of newly synthesized compounds, betulin derivatives, which could be potential drugs with anticancer activity. The two mobile phases were used for the purpose of experimental determination of lipophilicity for mono- and diesters investigated. The first mobile phase consists of acetate and Tris buffer, whilst the second consists of 1,4-dioxane and acetate buffer. The value of the retardation factor was recalculation into the *R*_M_ value, and then *R*_M0_ values were obtained by extrapolating acetone or 1,4-dioxane concentration to zero. The chromatographic data of lipophilicity were correlated with theoretically obtained values of ALOGPS, AClogP, miLogP, ALOGP, MLOGP, XLOGP2, and XLOGP3. The particular correlation equations were obtained. The cluster analysis was also used to find similarity between the esters investigated on the basis of the experimental or theoretical value of lipophilicity obtained. The good correlation between experimentally and theoretically calculated lipophilicity gives the possibility of prediction of this value on the basis of the correlation equation. On the basis of similarity analysis was stated strong connections between the structure and the value of lipophilicity, for both experimental and theoretical values.

## 1. Introduction

Betulin and its derivatives are lately the subjects of investigation taken into consideration by a lot of researches. The betulin has the cytotoxic activity against the melanoma cells and is more cytotoxic in rapidly dividing cells which is beneficial during chemotherapy treatment [1]. Betulin has also other biological activity, for example, exhibits protective action against the toxic effect of cadmium salts [2]. The activity of betulin and its derivatives with respect to the influenza virus of type A is also described [2]. The cytotoxicity of betulin-derived carbamates against the ovarian cell line was also stated [3]. Some heterocyclic betulin derivatives show the antiparasitic activity. They inhibit the growth of some amastigotes [4]. The other group of researchers synthesized some heterocyclic triterpenoids derived from betulin and betulinic acid [5]. The cytotoxicity to hematological tumors was observed. The antibacterial activity against five bacterial strains was also investigated and confirmed for semisynthetic betulin derivatives [6]. The compounds, betulin derivatives, analyzed in this work were synthesized, and their cytotoxic properties were estimated and described [7]. Also, the structure was confirmed by use of X-ray analysis in the DMSO solvate [8]. The other derivatives of betulin were synthesized, and their anticancer activity was analyzed by the same group of scientists [9, 10]. Thin-layer chromatography is widely used in pharmacy for analysis of pharmaceutical preparations and for determination of lipophilicity. Lipophilicity is also a very important factor, taking into account during designing a new drug. This is one of the most important and most effective physicochemical descriptors, widely used in the pharmaceutical industry as well. Lipophilicity has a big meaning and significant factor that determines the bioavailability [11]. The proper, optimum value of lipophilicity determines the solubility in biological fluids, the capacity of penetration through the biological membranes, and achieving the particular molecular structure [12]. The factor that determines the lipophilicity properties of the compounds is the intermolecular interactions between molecules of solute and solvent. There are many different theoretical and experimental methods that allow determining the partition coefficient. The popular and often-used method is the shake-flask method [11]. It is based on extraction in the octanol-water system [11]. The shake-flask method causes problems in the case of polar or highly lipophilic substance. It also requires the high purity of the substance analyzed and the solute [13]. There are also some chromatographic methods, very useful and popular for lipophilicity determination. Applying reversed-phase thin-layer chromatography or reversed-phase high-performance liquid chromatography, it is possible to measure the lipophilicity using an appropriate selection of mobile phases [11]. Another very important factor during designing new drug is QSAR (Quantitative Structure-Activity Relationships) analysis. Initially, QSAR analysis was used in toxicology. The relationships between toxicity and solubility in water of alcohols were then presented [14]. The aim of QSAR analysis is to settle the math, quantitative relationship, between the biological activity and the chemical structure of a series of compounds [11]. This is two-step analysis. The first step is descriptions of the molecular structure, and the second is multifactor correlation analysis between the molecular descriptors and the biological activity observed. The partition coefficient is very often used as a parameter describing the biological activity of the compounds investigated because it is strongly correlated with the ability of a substance to cross the biological membranes [14]. Some of our previous works take into consideration the relationships between the structure of newly synthesized compounds and biological activity expressed as partition correlation [15–17]. In all cases, the RP-TLC was used for determining the values of partition coefficients of the compounds investigated. The cluster analysis was also carried out. There was no relation between the structure and the experimental value of the partition coefficient (log *P*). The aim of this work was to determine the experimental and theoretical values of lipophilicity of new acetylene betulin derivatives. The relationships between these experimental and theoretical values of lipophilicity and the cluster analysis for all values of lipophilicity for new betulin derivatives were carried out for the purpose to define the relationships between the structure and lipophilic properties of new betulin derivatives. The usefulness of QSAR was showed.

## 2. Materials and Methods

### 2.1. Chemicals

**Figure 1:**
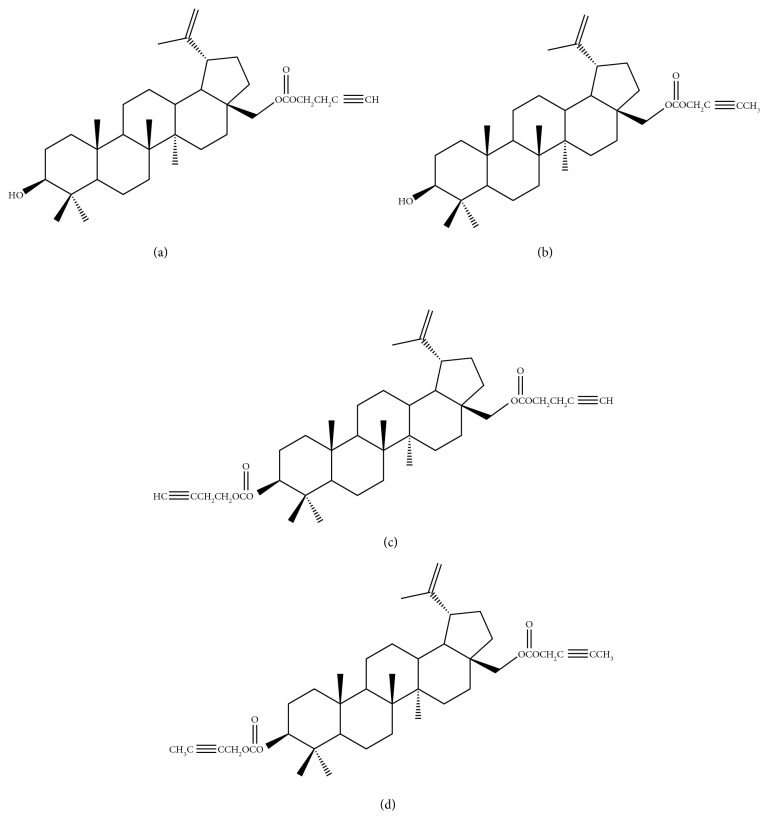
The structural formulas of newly synthetized mono- and diesters: (a) **1**; (b) **2**; (c) **3**; (d) **4**.

Synthesis of acetylenic derivatives of betulin was described previously [7]. The structure of all new compounds was determined on the basis of their ^1^H-NMR, ^13^C-NMR, IR, and MS spectra. All examined esters are presented in Figure 1.

### 2.2. Thin-Layer Chromatography

Partition chromatography was carried out on plates precoated with silica gel RP-18F_254_ (1.05559.0001, Merck, Germany). Plates were developed in a horizontal chamber previously saturated with vapor of the mobile phase. The mobile phases were as follows: mobile phase I—acetone (POCH; Gliwice, Poland) as the organic modifier and aqueous Tris buffer with pH 7.4 and ionic strength 0.2 M (Tris, Fluka, Niemcy), and mobile phase II—1,4-dioxane as the organic modifier and the solution of the acetate buffer (mixture of 0.2 M sodium acetate and 0.2 M acetic acid) with pH 4.8. Chemicals for mobile phase II were supplied by POCH (Gliwice, Poland). Contents of the organic modifier in the mobile phase ranged from 10 to 35 in 5% increments. The solutions of individual compounds were spotted in triplicate for each compound. Spots obtained were visualized by spraying the chromatographic plates with 10% sulphuric acid in ethanol and then drying in temperature of 110°C for 5 minutes. The average value of *R*_F_ was calculated and converted into the *R*_M_ value according to the following equation:

(1) $$\begin{array}{c} R_{\text{M}}=\log\left[\left(\frac{1}{R_{\text{F}}}\right)-1\right], \end{array}$$

*R*_M_ values were linearly dependent on acetone contents in a mobile phase. *R*_M0_ values were obtained by extrapolating acetone or 1,4-dioxane concentration to zero, according to the following equation:

(2) $$\begin{array}{c} R_{\text{M}}=R_{\text{M0}}+bC, \end{array}$$

where *C* is the concentration of acetone or 1,4-dioxane (%, v/v) in the mobile phase and *b* is the regression term.

### 2.3. Computational Software

The procedures of ALOGPS, AClogP, miLogP, ALOGP, MLOGP, XLOGP2, and XLOGP3 were used to calculate the lipophilicity of betulin derivatives **1**–**4**. The calculation procedures have been taken from the Internet database as in the case of our previous work [15, 18, 19]. The following calculation methods were used to calculate the values of the individual partition coefficients:

- ALOGPs: the method was developed on the basis neutral network ensemble analysis of more than 12000 organic compounds from the Physprop database using 75 E-state indices [20, 21]
- AClogP: the atom-additive method considering 369 atom-type-based contribution values [22]
- miLogP: the method for log *P* prediction developed at Molinspiration is based on group contributions, by fitting calculated log *P* with experimental log *P* for a training set more than 12000, mostly drug-like molecules [23]
- ALOGP: atomic contribution approach applying to neutral organic compounds containing among other N and halogen atoms [24, 25]
- MLOGP: Moriguchi octanol-water partition coefficient is based on quantitative structure—log *P* relationships, by using topological indexes [26]
- XLOGP2: additive atom/group model which uses 90 basic atom types [27]
- XLOGP3: knowledge-based approach based on the additive atom/group model which starts from the known log *P* value of a similarly reference compound [27]

### 2.4. Correlation and Cluster Analysis

On the basis of results of lipophilicity obtained (experimental and theoretical), the correlation and cluster analysis were performed. The analysis was carried out by use of STATISTICA 13.1 software.

## 3. Results and Discussion

Two mobile phases were considered: acetone-Tris and 1,4-dioxane-acetate buffer. The values of log *P*_TLC_ were calculated on the basis of known values of *R*_M0_, which were obtained by use of chromatographic analysis. First of all, the calibration curve was prepared for both mobile phases. The standard substances were as follows: acetanilide, 4-bromoacetophenone, benzophenone, anthracene, and DDT (dichlorodiphenyltrichloroethane). The values of *R*_M0_ for these substances, for both mobile phases, were taken from the literature [16, 28, 29].

First, the mobile phase: acetone-Tris buffer, was considered. The correlation between the literature log *P*_lit_ and experimentally received *R*_M0_ parameters for standard substances was described by the following equation:

(3) $$\begin{array}{c} \log\;P_{\text{TLC}}\;\;=1.1683R_{\text{M0}}+0.3778\left(r=0.995;\text{SD}=0.228\right). \end{array}$$

Tables 1 and 2 present the values of literature lipophilicity of standard compounds and the experimental values of lipophilicity for both standard and investigated compounds.

Then, the mobile phase 1,4-dioxan-acetate buffer was taken into consideration as well.

The correlation between the literature log *P*_lit_ and experimentally received *R*_M0_ parameters for standard substances was described by the following equation:

(4) $$\begin{array}{c} \log\;P_{\text{TLC}}=1.0715R_{\text{M0}}+0.7311\left(r=0.993;\text{SD}=0.269\right). \end{array}$$

Tables 3 and 4 present the values of literature lipophilicity of standard compounds as well as the experimental values of lipophilicity for both standard and investigated compounds.

Table 5 presents the values of theoretical lipophilicity for compounds investigated **1**–**4**, calculated by computer software available on the Internet database [18, 19].

The correlation analysis was done between the experimental and theoretical values of lipophilicity for compounds investigated, for both mobile phases used.  Table 6 presents the correlation coefficients for mobile phase I and Table 7 for mobile phase II.

In the case of mobile phase I which consists of acetone and Tris buffer, the highest value of the correlation coefficient (0.999) has the relationship between the experimentally determined log *P*_TLC_ and theoretically calculated XLOGP3 and the lowest value of the correlation coefficient (0.870) has the relationship between the experimentally determined log *P*_TLC_ and theoretically calculated ALOGPs.

In the case of mobile phase II which consists of 1,4-dioxane and acetate buffer, the highest value of the correlation coefficient (0.968) has the relationship between the experimentally determined log *P*_TLC_ and theoretically calculated XLOGP2 and the lowest value of the correlation coefficient (0.841) has the relationship between the experimentally determined log *P*_TLC_ and theoretically calculated miLogP.

Must be underlined that even though the values of correlation coefficients for mobile phase II are a little lower than that for mobile phase I, for both phases they are relatively high and not lower than 0.841. The correlation analysis shows the strong connection between the structure and the values of lipophilicity because the values of lipophilicity taken from the Internet database are mostly based on the structural properties. It gives a possibility to use the theoretically calculated values of lipophilicity during designing the new compounds with biological activity that required a particular value of this property.

Tables 8 and 9 present all correlation parameters for relationships between the experimental values of lipophilicity (log *P*_TLC_) and those theoretical values (ALOGP_S_, AClogP, miLogP, ALOGP, MLOGP, XLOGP2, and XLOGP3) for analysis by use of mobile phases I and II. These relationships described by correlation equations can be useful for prediction of experimental values of lipophilicity for new mono- and diesters.

The cluster (similarity analysis) was also carried out for all data obtained, separately for each mobile phase, taking into consideration both experimental (log  *P*_TLC_) and theoretical (ALOGPS, AClogP, miLogP, ALOGP, MLOGP, XLOGP2, and XLOGP3) values of lipophilicity.

**Figure 2:**
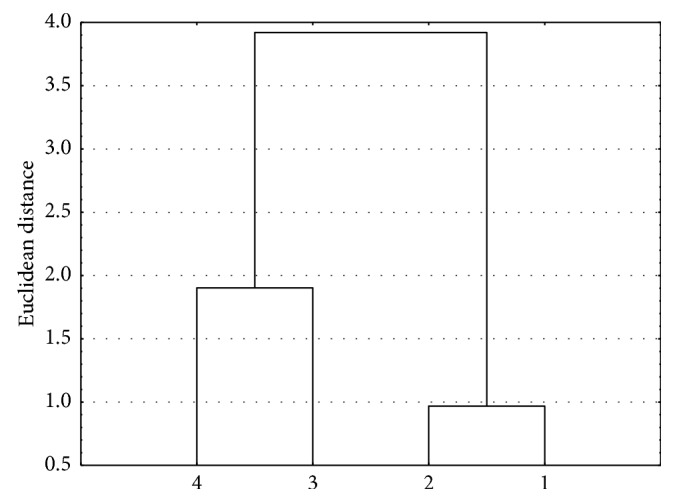
Similarity analysis for the experimental and theoretical values of the lipophilicity for the compounds investigated and mobile phase I (acetone-Tris buffer).

**Figure 3:**
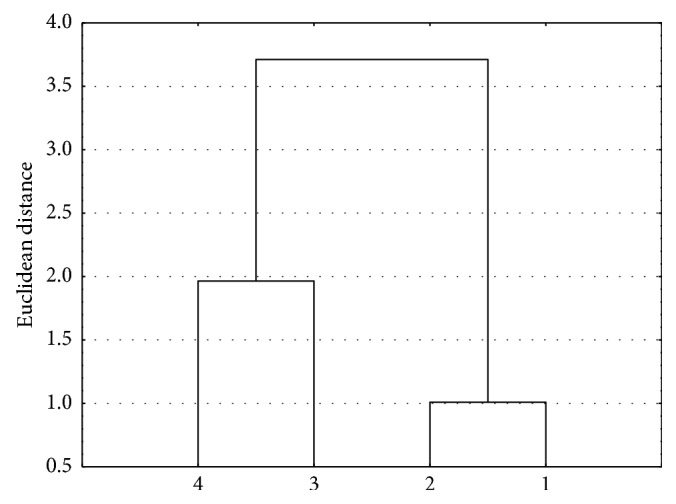
Similarity analysis for the experimental and theoretical values of the lipophilicity for the compounds investigated and mobile phase II (1,4-dioxane-acetate buffer).

Figures 2 and 3 present consequently analyses that were done.

In both cases of cluster analysis, two clusters are formed. The first cluster consists of diesters **3** and **4**, and the second one consists of monoesters **1** and **2**. Diesters **3** and **4** have a complex substituent at the carbon 3 of the basic structure of betulin, whilst in the case of esters **1** and **2**, there is the hydroxylic group. This similarity analysis shows the strong connection between the structure and the lipophilicity for compounds investigated. The grouping of the compounds investigated shows the connection between the structure and the values of lipophilicity in the case of newly synthesized mono- and diesters. It could be used for predicting the lipophilicity during designing the new compounds with biological activity, used potentially as drugs.

The other new biologically active compounds were investigated previously, and not always the good correlation between the structure and the lipophilicity was stated. For example, this relationship for newly synthesized quinobenzothiazines gave very low values of correlation coefficients [15, 17]. It means that the computer software does not take into consideration the other interaction between the atoms and bond but only the structure. On the contrary, analysis of the lipophilicity of 5,8-quinolinedione compounds showed, as in the case of the compound analyzed in this work, strong connections between experimental and theoretically calculated values of lipophilicity, that can serve to predict these values during designing new drugs [16].

## 4. Conclusion

Designing the new compounds with biological activity is nowadays very important because of constantly growing needs for new drugs. Researches all the time take an effort to design more and more compounds with a proper structure in order to obtain the substance with particular biological activity and with the most optimal value of lipophilicity. The new mono- and diesters presented in this work have the biological activity, for example, against P388 and CCRF/CEM leukemia cell lines [7]. The good correlation between experimentally and theoretically calculated lipophilicity gives the possibility of prediction of this value on the basis of the correlation equation. On the basis of similarity analysis was stated strong connections between the structure and the value of lipophilicity, for both experimental and theoretical values.

## Figures and Tables

**Table 1:** Literature (log *P*_lit_) and experimental (*R*_M0_ and log *P*_TLC_) lipophilicity values for standard compounds.

Compound	Log *P*_lit_	*R*_M0_	*b*	*r*	Log *P*_TLC_
Acetanilide	1.21	0.67	−0.01	0.975	1.16
4-Bromoacetophenone	2.43	1.92	−0.02	0.997	2.62
Benzophenone	3.18	2.47	−0.03	0.997	3.27
Anthracene	4.45	3.21	−0.04	0.987	4.13
DDT	6.38	5.21	−0.06	0.997	6.47

*b* is the slope, and *r* is the correlation coefficient for the linear relationship *R*_M_ = *R*_M0_ + *b*C.

**Table 2:** The experimental values of lipophilicity for investigated compounds **1**–**4** in the mobile phase acetone-Tris buffer.

Compound	*R*_M0_	*b*	*r*	Log *P*_TLC_
**1**	6.26	−0.07	0.990	7.68
**2**	6.31	−0.07	0.995	7.74
**3**	7.76	−0.08	0.994	9.43
**4**	7.97	−0.08	0.996	9.68

*b* is the slope, and *r* is the correlation coefficient for the linear relationship *R*_M_ = *R*_M0_ + *b*C.

**Table 3:** Literature (log *P*_lit_) and experimental (*R*_M0_ and log *P*_TLC_) lipophilicity values for standard compounds (mobile phase 1,4-dioxane: acetate buffer).

Compound	Log *P*_lit_	*R*_M0_	*b*	*r*	Log *P*_TLC_
Acetanilide	1.21	0.39	−0.01	0.909	1.15
4-Bromoacetophenone	2.43	1.49	−0.02	0.998	2.32
Benzophenone	3.18	2.36	−0.03	0.998	3.26
Anthracene	4.45	3.80	−0.04	0.987	4.80
DDT	6.38	5.02	−0.06	0.999	6.11

*b* is the slope, and *r* is the correlation coefficient for the linear relationship *R*_M_ = *R*_M0_ + *b*C.

**Table 4:** The experimental values of lipophilicity for investigated compounds **1**–**4** in the mobile phase 1,4-dioxane : acetate buffer.

Compound	*R*_M0_	*b*	*r*	Log *P*_TLC_
**1**	6.29	−0.07	0.988	7.46
**2**	6.01	−0.07	0.988	7.17
**3**	7.06	−0.08	0.993	8.29
**4**	7.57	−0.08	0.994	8.84

*b* is the slope, and *r* is the correlation coefficient for the linear relationship *R*_M_ = *R*_M0_ + *b*C.

**Table 5:** The theoretical values of lipophilicity for analyzed compounds **1**–**4**.

Compound	ALOGPs	AClogP	miLogP	ALOGP	MLOGP	XLOGP2	XLOGP3
**1**	6.23	6.94	8.06	8.85	6.40	9.15	9.84
**2**	6.74	7.50	8.47	8.48	6.40	9.38	9.89
**3**	7.14	8.05	8.67	11.38	6.75	10.49	11.39
**4**	8.20	9.16	9.11	10.64	6.75	11.16	11.49

**Table 6:** Values of coefficient for correlation analysis between the experimental and theoretical values of lipophilicity for compounds investigated and mobile phase acetone-Tris buffer.

	log *P*_TLC_	ALOGPs	AClogP	miLogP	ALOGP	MLOGP	XLOGP2	XLOGP3
log *P*_TLC_	1.000	0.870	0.891	0.871	0.942	0.995	0.977	0.999
ALOGPs		1.000	0.999	0.985	0.655	0.818	0.954	0.846
AClogP			1.000	0.988	0.689	0.844	0.966	0.870
miLogP				1.000	0.672	0.827	0.944	0.853
ALOGP					1.000	0.970	0.850	0.957
MLOGP						1.000	0.952	0.999
XLOGP2							1.000	0.966
XLOGP3								1.000

**Table 7:** Values of coefficient for correlation analysis between the experimental and theoretical values of lipophilicity for compounds investigated and mobile phase 1,4-dioxane-acetate buffer.

	Log *P*_TLC_	ALOGPs	AClogP	miLogP	ALOGP	MLOGP	XLOGP2	XLOGP3
Log *P*_TLC_	1.000	0.886	0.900	0.841	0.868	0.943	0.968	0.952
ALOGPs		1.000	0.999	0.985	0.655	0.818	0.954	0.846
AClogP			1.000	0.988	0.689	0.844	0.966	0.870
miLogP				1.000	0.672	0.827	0.944	0.853
ALOGP					1.000	0.970	0.850	0.957
MLOGP						1.000	0.952	0.999
XLOGP2							1.000	0.966
XLOGP3								1.000

**Table 8:** Correlation parameters for relationships between experimental (log *P*_TLC_) and theoretical (log *P*_calc_) values of lipophilicity for mobile phase I.

Software	*b*	*C*	*r*	SD
ALOGPS	1.1136	0.7512	0.870	0.65
AClogP	1.0074	0.6618	0.891	0.59
miLogP	2.1356	−9.6854	0.871	0.64
ALOGP	0.7229	1.5205	0.942	0.44
MLOGP	5.2714	−26.0271	0.995	0.13
XLOGP2	1.1059	−2.4765	0.977	0.28
XLOGP3	1.1741	−3.8748	0.999	0.07

*b* is the slope, and *r* is the correlation coefficient for the linear relationship log *P*_TLC_=*b* log *P*_calc_+*C*.

**Table 9:** Correlation parameters for relationships between experimental (log *P*_TLC_) and theoretical (log *P*_calc_) values of lipophilicity for mobile phase II.

Software	*b*	*C*	*r*	SD
ALOGPS	0.8105	2.2039	0.886	0.44
AClogP	0.7266	2.1909	0.900	0.41
miLogP	1.4748	−4.7099	0.841	0.51
ALOGP	0.4762	3.2557	0.868	0.46
MLOGP	3.5714	−15.5421	0.943	0.31
XLOGP2	0.1736	0.0787	0.968	0.24
XLOGP3	0.7998	−0.5798	0.952	0.29

*b* is the slope, and *r* is the correlation coefficient for the linear relationship log *P*_TLC_=*b* log *P*_calc_+*C*.

## Data Availability

All chromatographic data used to support the findings of this study are available from the corresponding author upon request.
